# Semaphorins in Adult Nervous System Plasticity and Disease

**DOI:** 10.3389/fnsyn.2021.672891

**Published:** 2021-05-11

**Authors:** Daniela Carulli, Fred de Winter, Joost Verhaagen

**Affiliations:** ^1^Laboratory for Neuroregeneration, Netherlands Institute for Neuroscience, Royal Academy of Arts and Sciences, Amsterdam, Netherlands; ^2^Department of Neuroscience Rita Levi-Montalcini and Neuroscience Institute Cavalieri Ottolenghi, University of Turin, Turin, Italy

**Keywords:** semaphorins, plasticity, perineuronal net, schizophrenia, epilepsy, Alzheimer’s disease, multiple sclerosis, autism

## Abstract

Semaphorins, originally discovered as guidance cues for developing axons, are involved in many processes that shape the nervous system during development, from neuronal proliferation and migration to neuritogenesis and synapse formation. Interestingly, the expression of many Semaphorins persists after development. For instance, Semaphorin 3A is a component of perineuronal nets, the extracellular matrix structures enwrapping certain types of neurons in the adult CNS, which contribute to the closure of the critical period for plasticity. Semaphorin 3G and 4C play a crucial role in the control of adult hippocampal connectivity and memory processes, and Semaphorin 5A and 7A regulate adult neurogenesis. This evidence points to a role of Semaphorins in the regulation of adult neuronal plasticity. In this review, we address the distribution of Semaphorins in the adult nervous system and we discuss their function in physiological and pathological processes.

## Introduction

**FIGURE 1:**
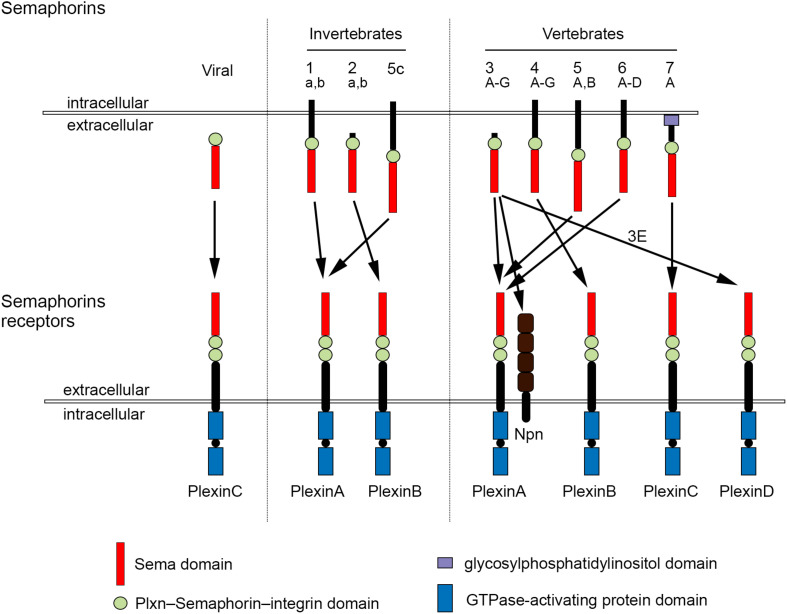
Semaphorins and their receptors. Semaphorins can be categorized into eight classes. Viral Sema is found in the genomes of certain DNA viruses; Sema1, Sema2, and Sema5c comprise the invertebrate Semaphorins; the other Semaphorin classes are found in vertebrates. Semaphorins are secreted (viral Sema, Sema2, and Sema3), membrane-spanning (Sema1, Sema4, Sema5, and Sema6) or glycosylphosphatidylinositol-anchored proteins (Sema7A). Semaphorins bind to Plxn receptors (PlxnA1–PlxnA4, PlxnB1–PlxnB3, PlxnC1, and PlxnD1) – see arrows for specific interactions. Sema3 require Npn for binding to PlxnA.

The development of complex tissues depends on proliferation, differentiation and migration of cells. Cell guidance cues regulate these events and continue to be essential throughout life to maintain tissue homeostasis. Semaphorins constitute a large family of cell guidance cues, which are present in some viruses and conserved across animal species, from worms and flies to humans. Thirty Semaphorin proteins have been identified so far. They can be divided into eight classes (Sema1-7 and the viral Semaphorins, SemaV) on the basis of phylogenetic relationships and structural features. Sema1, Sema2, and Sema5C are found in invertebrates, whereas all the other Semaphorin classes are found in vertebrates (Bamberg et al., 1999; Pasterkamp, 2012; Alto and Terman, 2017; Figure 1). Semaphorins can be secreted (Sema2, Sema3, and SemaV), membrane-spanning (Sema1, Sema4, Sema5, and Sema6) or glycosylphosphatidylinositol-anchored (Sema7A). The structural hallmark of the Semaphorin protein family is an extracellular domain at the N-terminal region, important for dimerization and interaction specificity, called Sema domain, which is followed by a Plxn–Semaphorin–integrin domain and by distinct protein domains that further define Semaphorins (Zhou et al., 2008; Figure 1).

First characterized by their ability to act as repulsive cues for growing neurites (Kolodkin et al., 1992, 1993; Luo et al., 1993), Semaphorins are now known to be crucial molecules also for the development and functioning of the musculoskeletal, cardiovascular, respiratory, immune, endocrine, reproductive, hepatic, and renal system. In addition, Semaphorin signaling has been linked to diseases affecting these systems, as well as to cancer (Roth et al., 2009; Neufeld et al., 2012; Pasterkamp, 2012; Tamagnone, 2012; Giacobini and Prevot, 2013; Kang and Kumanogoh, 2013; Kumanogoh and Kikutani, 2013).

The effects of Semaphorins occur through binding to their receptors, the neuropilin (Npn) and plexin (Plxn) protein families (Figure 1). Plxns are grouped in four classes, from A to D, with four A-type, three B-type, one C-type and one D-type. The Plxn extracellular region contains several sema domains, which are important for binding to Semaphorins, whereas the intracellular region contains GTPase-activating protein domains (Takahashi et al., 1999; Tamagnone et al., 1999). In general, Semaphorins exist as homodimers, both in an unbound state and when interacting with Plxns. Semaphorin homodimers bring together two Plxn monomers or disrupt existing Plxn homodimers, relieving Plxn autoinhibition, which might be caused by an interaction between the sema domain of Plxn and the rest of the Plxn extracellular domain (Takahashi and Strittmatter, 2001; Kong et al., 2016). Once activated, Plxn signals through downstream molecules, including GTPases of the Rho family, protein kinases such as MAPK, and enzymes such as MICAL (molecule interacting with casL), which induce the phosphorylation of intracellular proteins of the collapsin responsive mediator protein (CRMP) family (Vikis et al., 2000; Hu et al., 2001; Liu and Strittmatter, 2001; Terman et al., 2002; Pasterkamp et al., 2003; Hota and Buck, 2012). CRMPs, in turn, affect actin and microtubule dynamics (Hung et al., 2010, 2011; Alto and Terman, 2017). Membrane-associated Semaphorins can also act as receptors or co-receptors for Semaphorins located on other cells, a phenomenon known as reverse signaling (Battistini and Tamagnone, 2016).

Class 3 Semaphorins require Npn as co-receptors (Npn-1 and -2). Npn-1 homodimers function as ligand-binding receptors for Sema3A and Sema3D; Npn-2 homodimers as receptors for Sema3F; and Npn-1 and Npn-2 heterodimers as receptors for Sema3B, 3C, 3E, and 3G (He et al., 2019; Toledano et al., 2019). Npn are transmembrane proteins with short intracellular domains that lack intrinsic enzymatic or signaling activity. They do not seem to act as a direct bridge between Plxn and Semaphorins but may function in the presentation of Semaphorin to Plxn. In addition, Npn can bind vascular endothelial growth factor (VEGF) in co-receptor complexes with VEGF receptors (Kruger et al., 2005; Pasterkamp, 2012), regulating blood and lymphatic vessel growth (Tammela et al., 2005).

Additional receptors can directly bind Semaphorins, including CD72 (Kumanogoh et al., 2000), Tim2 (Kumanogoh et al., 2002), and integrins (Pasterkamp et al., 2003). Moreover, co-receptors that associate with Sema binding receptors affect the signaling outcome of Sema-receptor interactions (Sharma et al., 2012). Cell adhesion molecules, such as Nr-CAM and L1 CAM can associate with Npn receptors and can be required for transducing class 3 Sema signals (Castellani et al., 2000, 2004; Falk et al., 2005). In addition, a number of receptor tyrosine kinases, such as VEGF receptor 2, Met, ErbB2 and off-track, associate with Plxns and Npns and become transactivated upon Sema binding (Sharma et al., 2012). Interestingly, Semaphorin function can be modulated by binding to proteoglycans (Kantor et al., 2004; de Wit et al., 2005; Zimmer et al., 2010; Cho et al., 2012; Dick et al., 2013). For example, class 5 Semaphorins demonstrate axon repulsive properties on neurites that co-express chondroitin sulfate proteoglycans and Plxns, while they turn into attractive cues if neurites express heparan sulfate proteoglycans adjacent to Plxns (Kantor et al., 2004).

Semaphorins have been discovered in the early 1990s as repulsive axon guidance molecules, enabling axons to find their targets and thus contributing to nervous system development (Kolodkin et al., 1992, 1993; Luo et al., 1993). In the peripheral nervous system, Semaphorins of several classes form molecular boundaries to prevent axons of dorsal root ganglion neurons, cranial nerves, spinal motoneurons or sympathetic neurons from entering inappropriate areas (Masuda and Taniguchi, 2016). Repulsive Semaphorin signaling is also crucial in the control of axon pathfinding of several classes of central nervous system (CNS) neurons during development (Sahay et al., 2003; Kolk et al., 2009; Pignata et al., 2016; Alto and Terman, 2017; Okada et al., 2019). The main mechanism how Semaphorins act as guidance molecules is through activation of Plxn signaling, which induces cytoskeletal changes in the growth cone of developing axons, such as depolymerization of actin filaments, attenuation of microtubule dynamics, and collapse of microtubule arrays (Goshima et al., 1997; Fritsche et al., 1999; Hung et al., 2010).

In the last three decades, Semaphorins have been shown to be involved in many other developmental processes that shape the nervous system, including axon defasciculation (Kolodkin et al., 1992; Tran et al., 2007; Claudepierre et al., 2008; Pecho-Vrieseling et al., 2009; Imai, 2012; Assens et al., 2016), lamina-specific patterning of synaptic connectivity (Skutella and Nitsch, 2001; Pasterkamp, 2012; Xie et al., 2019), axon terminal branching (Bagnard et al., 1998; Bagri et al., 2003; Dent et al., 2004; Cioni et al., 2013; Jung et al., 2019), dendritic morphogenesis and arborization (Polleux et al., 2000; Fenstermaker et al., 2004; Vodrazka et al., 2009; Ng et al., 2013; Cheadle and Biederer, 2014; Yamashita et al., 2014; Danelon et al., 2020), synapse formation (Godenschwege et al., 2002; Morita et al., 2006; Paradis et al., 2007; Yamashita et al., 2007; Tran et al., 2009; Kuzirian et al., 2013; Inoue et al., 2018; McDermott et al., 2018), subcellular target recognition by specific axons (Telley et al., 2016), pruning (Bagri et al., 2003; Sahay et al., 2003; Faulkner et al., 2006; Low et al., 2008; Uesaka et al., 2014), and removal of ectopic synapses (O’Connor et al., 2009; Tran et al., 2009; Mohan et al., 2018, 2021).

Many excellent reviews have addressed the functions of Semaphorins during nervous system development. Here, we will provide an overview of the role of Semaphorins in adult CNS physiology and pathology, including the role of Sema3A in plasticity processes through its interaction with the extracellular matrix (ECM).

## Semaphorins in Adult Nervous System Physiology

Semaphorins are found in the nervous system not only during development but also in adulthood. Early studies showed that Sema3A mRNA expression persists in several discrete areas of the adult CNS and PNS (Luo et al., 1993; Giger et al., 1996, 1998; Pasterkamp et al., 1998; de Wit and Verhaagen, 2003). Since then, the role of Sema3A and other Semaphorins in the physiology of the adult nervous system has been progressively unveiled, pointing to a role of these axon guidance cues in the regulation of neuroplasticity.

### Class 3 Semaphorins in Perineuronal Nets

**FIGURE 2:**
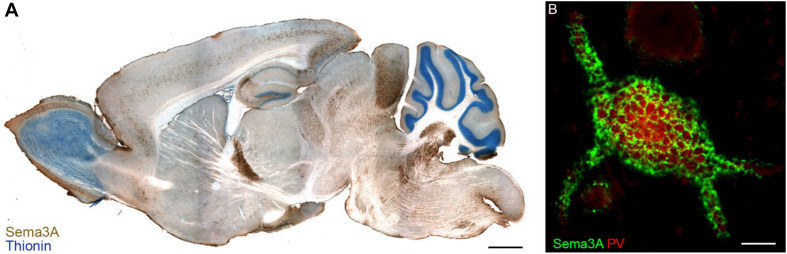
Sema3A in perineuronal nets in the adult mouse brain. Panel **(A)** shows a parasagittal section of the adult mouse brain in which Sema3A immunostaining (brown) is apparent throughout the whole rostro-caudal axis. In blue, thionin counterstaining. Reprinted with permission from Vo et al. (2013). **(B)** Sema3A (green) displays a lattice-like pattern, which is typical of perineuronal nets, around a parvalbumin (PV)+ neuron of the rat visual cortex. Scale bar: 2 mm **(A)**, 5 μm **(B)**.

Transcripts for Sema3A are found in distinct neuronal populations throughout the rostro-caudal axis of the adult CNS, as well as in meninges, pituitary gland and pineal gland, in rats as well in humans (Giger et al., 1998). Immunohistochemistry for Sema3A confirmed the expression of Sema3A in several regions of the adult rat and mouse CNS (Carulli et al., 2013; Vo et al., 2013; Giacobini et al., 2014; de Winter et al., 2016). Interestingly, Sema3A protein distribution can display different expression patterns. First, Sema3A can be diffusely localized in the neuropil. Secondly, Sema3A can be observed in or around myelinated axons. Thirdly, one of the more striking features of the anatomical distribution of Sema3A in the CNS is its accumulation in perineuronal nets (PNNs; Vo et al., 2013; Figure 2). PNNs are lattice-like aggregates of extracellular matrix molecules enwrapping the cell body and proximal dendrites of many types of CNS neuron. They form during postnatal development, contributing to the closure of critical periods for plasticity. The main scaffold of the PNN is composed by chondroitin sulfate proteoglycans (CSPGs), hyaluronan, link proteins and tenascin-R. Hyaluronan is a long unbranched polysaccharide, to which several CSPGs bind through their N-terminal domains, and this binding is strengthened by link proteins. CSPGs are cross-linked by Tenascin-R, which is a trimeric molecule which binds the CSPG C-terminal domain (Fawcett et al., 2019). The interaction of Sema3A with the PNN occurs through its binding to CSPG sugar chains (i.e., chondroitin sulfate glycosaminoglycans – CS-GAGs). The first indication of Sema3A interacting with CS-GAGs comes from the work by de Wit et al. (2005). Addition of CS-GAGs to the culture medium of Neuro2a cells (a murine neuroblastoma cell line) displaces cell surface bound Sema3A. Moreover, enzymatic removal of CS-GAGs using chondroitinase ABC releases Sema3A into the culture medium. Interestingly, injection of chondroitinase ABC *in vivo* abolishes Sema3A-labeling of PNNs which indicates that Sema3A is released from PNN following digestion of CSPGs (Vo et al., 2013). CS-GAGs consist of repeated disaccharide units made of glucuronic acid (GlcA) and N-acetylgalactosamine (GalNAc), and can present sulfate groups at various locations, resulting in extensive molecular heterogeneity. The most common CS-GAG isoform in the adult brain are: CS-A (C-4 sulfation on GalNAc), CS-C (C-6 sulfation on GalNAc), CS-D (C-2 sulfation on GlcA and a C-6 sulfation on GalNAc), and CS–E (C-4 and C-6 sulfation on GalNAc; Sugahara and Kitagawa, 2000). The interaction between Sema3A and PNNs is sulfation-dependent, with Sema3A interacting preferentially to CS-E extracted from adult brain PNNs (Dick et al., 2013) via two specific peptidic sequences located in the Sema3A C-terminal domain (Djerbal et al., 2019). Interestingly, one of these sequences is very similar to a sequence found in orthodenticle homeobox 2 (Otx2), which is involved in PNN formation and modulation of plasticity (Bernard and Prochiantz, 2016). This sequence mediates accumulation of Otx2 into PNNs, through binding to CS-GAGs (Beurdeley et al., 2012), raising the possibility that Sema3A and Otx2 could compete with one another for PNN binding. Interestingly, the lack of binding of Sema3A to CS-D (which is a disulfated CS-GAG structure, similar to CS-E, bearing two highly negatively charged sulfate groups) suggests that the interaction with CS-E is specific and is not due to a non-specific charge interaction (Dick et al., 2013). Recent data *in vivo*, however, show that the main CS types binding Sema3A are non-sulfated GalNAc residues at the non-reducing termini of CS-GAG chains (Nadanaka et al., 2020). Binding of Sema3A (and possibly other secreted Semaphorins) to CS-GAGs may be crucial for the presentation of Semaphorin to their receptors, and thus for the activation of Semaphorin signaling in specific brain locations.

The presence of Sema3A-positive PNN around neurons that do not express Sema3A mRNA (such as cortical interneurons) suggests that Sema3A is not necessarily produced by neurons endowed with a PNN, but it might be derived from nearby neurons or afferent ones. Indeed, in embryonic rat cortical neurons, Sema3A is actively transported in vesicles through the axon and dendrites of the cell. In axons, Sema3A is almost exclusively transported in an anterograde direction, and this transport is activity-dependent. Blocking action potentials results in an acceleration of Sema3A transport, whereas cell depolarization induces a transport arrest, which is accompanied by release of Sema3A at the cell surface (de Wit et al., 2006). Therefore, presynaptic neurons may produce Sema3A, transport it to their terminals, and subsequently deposit it in the PNN in an activity-dependent manner. In addition, Sema3A is highly expressed by meningeal cells (Giger et al., 1998; Niclou et al., 2003), suggesting that Sema3A is released by those cells into the cerebrospinal fluid and the parenchyma, traveling through the extracellular space from where it might be captured by PNNs. This is indeed what occurs to another PNN component, Otx2, in the adult brain. Otx2 is secreted from the choroid plexus and transported through the cerebrospinal fluid into the brain parenchyma. Here, it is captured by PNNs and then internalized in PNN-bearing neurons, where it acts as a transcription factor (Spatazza et al., 2013).

What is the function of Sema3A in PNNs? Sema3A in PNNs contributes to the end of the critical period in the visual cortex. Critical periods are time windows of intense brain development in which neuronal connections are highly plastic and shaped under the influence of environmental stimuli (Reh et al., 2020). Accumulation of Sema3A in PNNs in the rat visual cortex begins between postnatal day 28 and postnatal day 45, in coincidence with the closure of the critical period for ocular dominance plasticity. When rats are raised in darkness, plasticity persists into adulthood, and this is correlated with reduced levels of Sema3A-positive PNN structures. Notably, interfering with Sema3A signaling enhances ocular dominance plasticity in adult rats (Boggio et al., 2019). Overall, those data point to a role of perineuronal-Sema3A in closure of the critical period and repression of juvenile plasticity in the adult brain.

Another function of Sema3A may be related to restriction of structural plasticity, with Sema3A acting as an inhibitory cue for rewiring of existing connections or formation of new connections on PNN-bearing neurons. Indeed, when adult mice are reared in an enriched environment, a condition known to promote neuronal plasticity (Sale et al., 2014), a strong reduction in PNN CS-GAGs as well as Sema3A expression in PNN is observed in the cerebellar nuclei, in parallel with substantial remodeling of synaptic terminals (Foscarin et al., 2011; Carulli et al., 2013). Moreover, changes in Sema3A expression have been found during compensatory sprouting occurring after injury. Partial deprivation of cerebellar nuclei neurons of their main inputs, the Purkinje cells, results in a strong decrease of PNN and Sema3A labeling around denervated neurons, in association with structural reorganization of the local connectivity (Carulli et al., 2013). Overall, these studies suggest that Sema3A in PNNs is actively modulated to facilitate or restrict plasticity according to specific functional requirements.

In addition, Sema3A in PNN may affect molecular processes in the cell body of PNN-bearing neurons, as Sema3A receptors PlexinAs are detected on the soma of PNN+/PV+ neurons (Vo et al., 2013), and PV+ neurons in the visual cortex also express CRMP4 (Cnops et al., 2006). Here, we can speculate that Sema3A signaling may cause cytoskeletal changes, which may affect the distribution of post-synaptic channels and/or receptors, and thus synaptic plasticity and/or connectivity. Finally, Sema3A has been found to rigidify CS-E based matrices. Therefore, it may cross-link PNN-GAGs and thus contribute to PNN stability (Djerbal et al., 2019).

Another class 3 Semaphorin, Sema3B, shows a perineuronal net-like pattern (Vo et al., 2013), but its expression throughout the brain is more restricted than that of Sema3A. For example, whereas Sema3A in the hippocampal system is detected in the subiculum, CA1, CA2, CA3, and dentate gyrus, Sema3B-labeled PNNs are only observed in the subiculum. In the thalamic area, Sema3A labeling is associated with PNNs in the medial septal nucleus and the reticular thalamic nucleus, whereas Sema3B immunoreactivity is only found in the reticular thalamic nucleus (Vo et al., 2013). In the adult brain Sema3B mRNA is apparent in the choroid plexus (Kaiser and Bryja, 2020). It may thus be released into the cerebral spinal fluid and travel in the parenchyma extracellular space from where it could be incorporated into PNNs. Because Sema3B does not contain the PNN-binding peptide sequence shared by Sema3A and Otx2, it may interact with PNNs through differentially sulfated CS-GAGs, which may be found only on a small sub-population of PNNs.

### Homeostatic Synaptic Plasticity

Homeostatic plasticity is referred to all biological processes that neurons and neuronal circuits use to stabilize their activity around some set-point value in the face of perturbations, such as changes in cell size, synapse number or synapse strength, which would alter excitability (Turrigiano, 2008). An example of homeostatic plasticity is the increase in neurotransmitter release that follows an impairment in postsynaptic receptor function. Homeostatic plasticity is conserved from fly to humans (Davis, 2006). Recent evidence points to a role of Semaphorins in the control of homeostatic plasticity in the adult nervous system. For instance, at the neuromuscular junction (NMJ) in Drosophila, Semaphorin 2b is a muscle-derived, secreted signal that mediates the homeostatic control of presynaptic neurotransmitter release. After decreasing the amplitude of miniature excitatory postsynaptic potentials (by applying a glutamate-receptor antagonist), a significant increase in presynaptic neurotransmitter release occurs, which compensates for the postsynaptic perturbation and restores normal muscle excitation. This presynaptic homeostatic plasticity does not occur in Drosophila larvae containing a null mutation in the sema2b gene or the plxnB gene. Regulation of presynaptic homeostatic plasticity by Sema2b–PlxnB signaling occurs through the cytoplasmic protein Mical, which is present presynaptically and mediates actin depolymerization, which is necessary to release synaptic vesicles from the reserve pool and, thus, expand the readily releasable pool (Orr et al., 2017).

Another form of homeostatic plasticity is homeostatic scaling, which allows neurons to maintain their firing rates in the presence of changes in neuronal activity. For instance, blocking neuronal activity leads to increased synaptic strength, or upscaling, whereas increasing neuronal activity leads to decreased synaptic strength, or downscaling. Modulation of synaptic strength is largely dependent on distribution and function of postsynaptic receptors, such as AMPA receptors (Turrigiano, 2012). In cultured rat cortical neurons, elevating neuronal activity by bicuculline promotes Sema3F secretion, which contributes to a reduction in AMPA receptor number on the cell post-synaptic membrane. Indeed, no change in AMPA receptor has been found in Sema3F KO neurons. Thus, Sema3F, possibly by disrupting the interaction that exists between Npn-2 and AMPA receptors, mediates AMPA receptor downscaling following increased neuronal activity (Wang et al., 2017).

In conclusion, secreted semaphorins affect homeostatic plasticity by causing cytoskeleton modifications leading to increased release of pre-synaptic vesicles, or by attenuating the binding between Semaphorin receptors and post-synaptic neurotransmitter receptors, thereby affecting channel localization, trafficking, or biophysical properties.

### Hippocampal Plasticity, Learning and Memory

The adult hippocampus shows a remarkable capacity for changes in synaptic activity and structural reorganization during learning and memory. Modifications in synaptic strength, dendritic complexity and synapse number have been proposed to underlie memory processes (Leuner and Gould, 2010). Secreted Semaphorins, and particularly Sema3A, Sema3F, and Sema3G, are important players in the control of synaptic and structural plasticity in the adult hippocampus. In the work by Bouzioukh et al. (2006), exogenous application of Sema3A in hippocampal acute slices induces a striking reduction of pre- and post-synaptic excitatory puncta, and a strong depression of excitatory synaptic transmission in CA1 neurons, which are mediated by extracellular signal-regulated kinase (ERK) activation. Because Npn-1 is shown to be present in presynaptic terminals, Sema3A-induced depression of synaptic transmission could be the consequence of initial structural changes in presynaptic elements, which may then change the size of the synaptic cleft, reducing transmitter concentrations at postsynaptic receptors. Alternatively, Sema3A may destabilize the presynaptic membrane, causing then the retraction of the postsynaptic membrane (Bouzioukh et al., 2006).

Sema3A mRNA *in vivo* is shown to be expressed by stellate cells of the entorhinal cortex. They project to dendrites of granule cells, which are located in the molecular layer of the dentate gyrus and express Npn-1, but not Sema3A, transcripts (Giger et al., 1998). Induction of epileptic seizures in rats leads to a downregulation of Sema3A expression, which is correlated with aberrant sprouting of granule cell axons into the molecular layer of the dentate gyrus, resulting in the formation of a recurrent excitatory network (de Wit and Verhaagen, 2003; Holtmaat et al., 2003). These results suggest that Sema3A may be involved in constraining structural changes in the adult hippocampus.

Sema3F modulates the morphology and function of synapses in the adult hippocampus, but it has opposite effects from Sema3A. Sema3F mRNA is abundantly expressed in adult granule cells of the dentate gyrus and pyramidal neurons of CA1 and CA3 regions (Giger et al., 1998; Hirsch et al., 1999; Holtmaat et al., 2002; Bagri et al., 2003; Barnes et al., 2003). Sema3F has been shown to play a crucial role in modulating hippocampal basal synaptic transmission. Incubation of acute hippocampal slices with recombinant Sema3F induces an increase in the frequency and amplitude of miniature EPSCs in granule cells and CA1 pyramidal neurons. These effects may occur through binding of Sema3F to Npn-1, which is present in granule cells axons (mossy fibers) and infrapyramidal tract axons and terminals (Sahay et al., 2005). Furthermore, mice lacking Sema3F are prone to seizures (Sahay et al., 2005), suggesting that Sema3F is essential for the normal functioning of hippocampal circuits.

Sema3G is secreted by the vascular system in the CNS and has been shown to be essential for maintenance of fear memories. Mice lacking Sema3G in vascular endothelial cells display a significant impairment in retrieval of fear memories, and this is accompanied by a strong decrease in hippocampal LTP, in frequency of miniature excitatory postsynaptic currents in CA1 pyramidal neurons, and in number of spines on pyramidal neurons. Sema3G effects are mediated by Npn-2/PlxnA4 signaling and Rac1 activation in the postsynaptic compartment (Tan et al., 2019). These data indicate that Sema3G is essential for the control of neural circuit stability and cognitive functions exerted by endothelial cells through intercellular communication with neurons in the hippocampus.

Another Semaphorin, Sema4C, plays an important role in hippocampal plasticity and memory. Sema4C and its receptor PlxnB2 are expressed in the dentate gyrus, CA1 and CA3 regions of the adult mouse and are upregulated upon fear conditioning. When Sema4C signaling is disturbed in adult forebrain excitatory neurons, mice show a dramatic impairment in their ability to retain both recent and remote fear memories, pointing to a role of Sema4C-PlxnB2 signaling in the process of consolidation of this type of memory (Simonetti et al., 2019). Moreover, while normally fear conditioning is accompanied by an increase in dendritic complexity and spine number on CA1 pyramidal neurons (Trabalza et al., 2012; Abate et al., 2018), no anatomical changes are observed in the absence of functional Sema4C-PlxnB2 signaling (Simonetti et al., 2019). In addition, both inducible PlxnB2 knock-out mice and mice expressing a PlxnB2 loss-of-function mutant for the RhoA pathway display a reduced number of glutamatergic synapses and an increased number of GABAergic synapses in the hippocampus in naïve conditions. Overall, Sema4C-PlxnB2, via RhoA signaling, is involved in the maintenance of a stable synaptic connectivity in the adult hippocampus (Simonetti et al., 2019).

### Neurogenesis

Replacement of neurons by generation of new neurons (neurogenesis) does not occur in the adult mammalian brain with the exception of two regions: the subgranular zone (SGZ) of the hippocampal dentate gyrus and the subventricular zone (SVZ) of the lateral ventricles. These neurogenic regions contain neural stem cells (NSCs), which continuously provide newborn neurons to replace those in existing circuits. In the SGZ, NSCs give rise to intermediate progenitor cells, which generate neuroblasts. Neuroblasts will develop into dentate granule neurons. NSCs in the SVZ give rise to transient amplifying progenitors, which become neuroblasts. Neuroblasts will form a chain and migrate into the olfactory bulb, where they differentiate into interneurons (Obernier and Alvarez-Buylla, 2019). Semaphorins control different aspects of adult NSC development. In the hippocampus, Sema7A has been shown to suppress NSC proliferation at early stages, and to regulate dendrite growth and spine development of granule cells at later stages. Interestingly, control of NSC proliferation is mediated by PlxnC1, whereas dendritic regulation relies on integrin receptors (Jongbloets et al., 2017). Another Semaphorin involved in morphological maturation of adult born granule cells is Sema5A. Sema5A-PlxnA2 signaling prevents the formation of supernumerary dendritic spines on adult-born granule cells (Duan et al., 2014).

In the adult SVZ, Sema3A is expressed in both migratory neuroblasts and NeuN−positive mature neurons, where it inhibits the proliferation of NSCs and enhances neuronal differentiation (Sun et al., 2016).

In addition, it has been shown that PlxnB2 is expressed by neuroblasts in the adult SVZ. Knock-out mice for PlxnB2 show reduced neuroblast proliferation, faster migration and accelerated transition from tangential to radial migration (Saha et al., 2012). Because Sema4C is expressed in the olfactory bulb and along the rostral migratory stream (Wu et al., 2009), a classic Semaphorin/PlxnB2 repulsive mechanism may prevent neuroblasts from prematurely leaving the rostral migratory stream.

### Target Re-innervation in the Taste System

In the adult taste system, Semaphorins maintain their role of guidance molecules to ensure specific target innervation of adult nerve fibers. Taste receptor cells turn over continuously throughout life and, as a consequence, functional connections between existing ganglion axons (fibers of the VII, IX and X cranial nerves) and newly born taste receptor cells need to be continuously established. Each taste quality (sweet, bitter, sour, salty and umami) is encoded by a unique population of taste receptor cells, which is innervated by a matching set of ganglion neurons. Therefore, when new taste receptor cells are produced, they must express instructive cues to establish proper connectivity. Interestingly, among these cues are Sema3A and Sema7A, which are expressed by bitter and sweet taste receptor cells, respectively. Experiments employing conditional loss-of-function or gain-of-function approaches demonstrated that Sema3A or Sema7A mediate the connectivity between receptor cells coding a specific taste modality and their partner ganglion neurons, which are endowed with specific Sema receptors (Lee et al., 2017). Therefore, Semaphorins play a crucial role in allowing the taste system to maintain fidelity of signaling despite turnover of receptor cells.

### Target Re-innervation in the Olfactory System

The olfactory epithelium contains olfactory sensory neurons (OSNs) and supporting sustentacular cells (Farbman, 1994). OSN axons form the olfactory nerve and project to the olfactory bulb (OB), where they contact the dendrites of mitral, tufted and juxtaglomerular neurons in globular structures called glomeruli (Mombaerts, 1996). In most animals, the olfactory epithelium is a site of continual adult neurogenesis. Olfactory neurogenesis occurs throughout life in response to normal turnover, as mature OSNs have a limited lifespan of approximately 30–90 days (Graziadei et al., 1979). New OSNs derive from a population of precursor cells in the olfactory epithelium and are able to re-innervate their target, establishing functional synaptic contacts (Oley et al., 1975; Jane Roskams et al., 1994). During development Sema3A mRNA is expressed by OSNs and olfactory ensheathing cells in the nerve layer of the ventral OB (Giger et al., 1996; Schwarting et al., 2000; Williams-Hogarth et al., 2000). Sema3A may be secreted by OSNs into the local epithelial environment, where it may inhibit their own axons from reentering the epithelium and autosynapsing (Williams-Hogarth et al., 2000). In the ventral midline of the OB, Sema3A acts as a repulsive guidance cue for Npn-1+ axons, which avoid the ventral OB and instead project to either medial or lateral targets. Indeed, in Sema3A knock-out mice, many Npn-1+ axons are misrouted (Schwarting et al., 2000, 2004). Sema3F is secreted by early arriving OSN axons and is deposited in the dorsal OB, where it repels late-arriving OSN axons that express Npn-2 (Takeuchi et al., 2010). The expression of Sema3A in OSNs is gradually down-regulated during development. However, in the adult, Sema3A is up-regulated after unilateral lesioning of the OB, a manipulation that induces increased OSN neurogenesis, and there is a temporal correlation between Sema3A expression and the generation of new OSNs. Interestingly, Sema3A levels decline when the axons of regenerating neurons reach the region of their ablated target (Williams-Hogarth et al., 2000). These results suggest that Sema3A plays a role in guiding OSN axons out of the epithelium and to the OB not only during development but also during adult regeneration. It would be interesting to interfere with the expression/signaling of Sema3A to elucidate its role in the process of reinnervation of existing targets.

### Control of Gonadotropin Release

In recent years it has been discovered that Semaphorins, particularly Sema3A and Sema7A, play a pivotal role in orchestrating the control of reproduction in the hypothalamus. The reproductive cycle of mammals is regulated by hypothalamic gonadotropin-releasing hormone (GnRH) neurons. In females, GnRH neurons periodically extend their axons in the median eminence toward the pituitary portal circulation, into which they release GnRH. GnRH is then carried into the anterior pituitary, where it stimulates the release of gonadotropins (luteinizing hormone and follicle stimulating hormone), which in turn act on peripheral reproductive organs to regulate the estrous cycle. When GnRH-secreting axon terminals are distant from the pericapillary space of the median eminence, the access of the neurohormone to the pituitary portal circulation is impeded, and gonadotropin levels in the bloodstream are low. However, at the onset of the preovulatory surge, when GnRH has to be released to trigger ovulation, GnRH-secreting axon terminals undergo extensive axonal growth toward the vascular wall. Specialized ependymal cells called tanycytes enwrap the terminals of GnRH neurons, insulating the pericapillary space of pituitary portal vessels from GnRH nerve terminals. Interestingly, tanycytes show fluctuating expression of Sema7A during the estrous cycle. Sema7A induces the retraction of GnRH nerve terminals as well as the expansion of tanycytic processes, therefore hampering the access of GnRH axons to the portal vasculature (Parkash et al., 2015). In addition, vascular endothelial cells of the pituitary portal system express Sema3A. In contrast to Sema7A, Sema3A, released by endothelial cells during proestrus, promotes the growth of GnRH axons toward the vascular plexus (Giacobini et al., 2014).

### Semaphorin Expression in Adult Retina, Striatum and Cortex

A bulk of evidence revealed important roles for Semaphorins in retina lamination and circuit assembly during development (Matsuoka et al., 2011a, b, 2012). However, the function of Semaphorins in the adult retina is still poorly characterized. The expression of mRNAs for all class-3 Semaphorins and their receptor components is apparent in the rat retina during postnatal development and persists well into adulthood. The highest expression is found in retinal ganglion cells, whereas lower transcript levels are detected in different cell types in the inner nuclear layer (de Winter et al., 2004), which contains the cell bodies of horizontal, bipolar, amacrine cells, and Muller glia cells. Based on evidence from other CNS regions, it can be hypothesized that, once secreted within the retina or from retinal ganglion cells into retino-recipient areas in the brain, Semaphorins may contribute to the maintenance of established connections.

The striatum of the adult mouse shows high levels of expression of Npn-2, which is localized in the soma and dendrites of spiny projection neurons, as well as in axon terminals of cortical pyramidal neurons. Inducible deletion of Npn-2 in cortical pyramidal neurons in adult mice leads to increased spine numbers on those neurons, alteration of corticostriatal short-term plasticity, and impairment of striatum-dependent motor skill learning (Assous et al., 2019). This suggests that Npn-2 signaling, likely mediated by Sema3F, is essential for the maintenance and function of the adult corticostriatal circuitry.

While many studies unveiled the role of Semaphorins in cortical development during both embryonic and postnatal life (Polleux et al., 1998; Canty and Murphy, 2008; Chen et al., 2008; Bribián et al., 2014; Carcea et al., 2014), the function of Semaphorins in the adult neocortex has only been partially studied. As mentioned above, Sema3A in PNNs around PV+ neurons of the visual cortex restricts visual cortex plasticity (Boggio et al., 2019). In addition, Sema3E is reported in adult excitatory and inhibitory neurons of layers V and VI, but in monkeys it is found only in a subpopulation of excitatory layer VI neurons. In both species, the mRNA for PlxnD1, the receptor for Sema3E, exhibits a complementary lamina pattern (Watakabe et al., 2006). However, no functional data are available for a role of Sema3E-PlxnD1 signaling in adult cortical functions, but we can speculate that it may control the maintenance of lamina-specific synaptic connectivity in the cortex.

### Semaphorins and Myelin

In addition to the plethora of roles played in neuron development and formation of neuronal connections, class 3 Semaphorins, as well other classes of Semaphorins, control oligodendrogenesis. Oligodendrocytes develop from NSC−derived oligodendrocyte precursor cells (OPCs), which are generated in the ventricular zones of the embryonic neural tube and then migrate throughout the CNS. During rodent CNS development, OPCs are mostly generated during the first few postnatal weeks. After reaching their final destination, OPCs differentiate into mature oligodendrocytes. They wrap around neighboring axons, forming myelin sheaths, which are essential for the rapid and efficient conduction of electrical impulses along axons, as well as for preserving axonal integrity (Richardson et al., 2006; Butt et al., 2019). *In vitro* work by Cohen et al. (2003) shows that OPCs isolated from postnatal rat brainstem express several secreted and membrane-bound Semaphorins as well as Npn receptors. By using a stripe assay, they demonstrate that class 3 Semaphorins (Sema3A, B, C, F) inhibit the migration of OPCs, redirect OPC process outgrowth and cause OPC growth cone collapse, suggesting that Sema3s are repulsive guidance cues for OPCs during their migration through the CNS. The role of Sema3A as repulsive cue for OPCs has been confirmed *in vivo* in the optic nerve. The embryonic optic nerve is colonized by OPCs (Raff et al., 1983; Skoff, 1990), which migrate in a chiasmal-to-retinal direction (Small et al., 1988; Spassky et al., 2002). In a functional migration assay, Sema3A acts as a repulsive signal for OPCs migrating into the optic nerve, whereas Sema3F acts as an OPC attractive signal. Consistently with those observations, Sema3A is found to be produced by cells of the perineural mesenchyme, which surrounds the nerve, while Sema3F is expressed by retinal cells (Spassky et al., 2002). OPCs express Npn-1 and -2, as well as PlxnAs (Spassky et al., 2002; Okada et al., 2007; Piaton et al., 2011). Interestingly, expression of Npn and Plxn receptors, with the exception of PlxnA4, persists in adult OPCs throughout the CNS (Okada et al., 2007; Piaton et al., 2011), but their role in adult OPC physiology is not known.

Axon myelination relies onto an exact matching between the number of oligodendrocytes and the number and lengths of axons (Barres and Raff, 1999). During normal development, many more oligodendrocytes than needed are produced. Subsequently, a selection occurs, which results in the deprivation of excess oligodendrocytes by apoptosis (Barres et al., 1992; Trapp et al., 1997). Sema4D is part of a regulatory mechanism underlying the maintenance of the appropriate number of mature oligodendrocytes and myelin sheaths. In the mouse CNS, Sema4D is expressed in oligodendrocytes in all major fiber tracts, from the olfactory bulb and the corpus callosum to the spinal cord, at the time when they start colonizing the prospective white matter to form myelin (Moreau-Fauvarque et al., 2003). In Sema4D deficient mice, the number of mature oligodendrocytes is significantly increased, while the number of OPCs is not affected (Taniguchi et al., 2009; Yamaguchi et al., 2012), suggesting that Sema4D may act as an intrinsic inhibitory regulator of oligodendrocyte differentiation by promoting apoptosis. Although Sema4D expression in the CNS is generally decreased after 1 month of age, it is still apparent in adult oligodendrocytes (Moreau-Fauvarque et al., 2003). The receptor for Sema4D, PlxnB1, is highly expressed in axons of mature neurons (Worzfeld et al., 2004; Fazzari et al., 2007; Foscarin et al., 2009), pointing to a role of Sema4D signaling in stabilizing myelin-axon interaction. Indeed, during axonal sprouting of adult mouse Purkinje cells, PlxnB1 receptors are withdrawn from the membrane of neuritic segments where sprouting occurs, in concomitance with retraction of myelin sheath (Gianola and Rossi, 2004; Foscarin et al., 2009). These data suggest that Sema4D signaling may help stabilize myelin-axon interaction in adult Purkinje cells, which in turn may be important for restricting aberrant axon growth.

Another class 4 Semaphorin implicated in OPC migration and differentiation is Sema4F (Armendáriz et al., 2012). Both OPCs and oligodendrocytes express Sema4F. Incubation of optic nerve explants with conditioned media from Sema4F-transfected 293T cells reduces the outward migration of OPCs, without affecting proliferation. Conversely, incubation with the anti-Sema4F antibodies results in increased OPC migration. These data suggest that Sema4F contributes to the correct migration of OPCs along the optic nerve, ensuring no dispersion of cells or intermingling between them. In addition, when OPCs derived from neonatal rat brain are exposed to Sema4F, OPC differentiation is increased, as shown by the increased percentage of myelin basic protein expressing cells (Armendáriz et al., 2012). Thus, Sema4F would control not only the migration of precursors but also their differentiation into myelinating oligodendrocytes.

The timing of oligodendrocyte differentiation and myelination is regulated by Sema6A (Bernard et al., 2012). Oligodendrocytes express increasing levels of Sema6A mRNA between P0 and P15. Later on, the number of Sema6A+ oligodendrocytes decrease, although a few cells still express Sema6A mRNA in the adult white matter. In Sema6A-deficient mice, the differentiation of oligodendrocytes is delayed, but in the adult age the expression of myelin genes and the number and appearance of the nodes of Ranvier are similar to controls. This suggests that the lack of Sema6A is compensated by other molecular cues, such as Sema6B (Cohen et al., 2003; Bernard et al., 2012).

Although most of our knowledge on Semaphorins still comes from development, recent evidence point to an involvement of Semaphorins in many physiological functions of the adult brain, from control of plasticity and memory to regulation of adult NSC proliferation and migration (Table 1). The importance of Semaphorins for brain physiology is also corroborated by the evidence that several brain diseases are associated with alterations in Semaphorin expression or Semaphorin signaling, which, depending on the disease, may impact brain function during development or in adulthood. This topic is discussed in the following section.

**TABLE 1:** Overview of the main known functions of Semaphorins in the adult brain.

Semaphorin	Function	References
Sema3A	Restriction of visual cortex plasticity Inhibition of SVZ neurogenesis Specific re-innervation of bitter taste receptor cells Control of axon growth of GnRH neurons Affecting the rigidity of PNN	Boggio et al. (2019) Sun et al. (2016) Lee et al. (2017) Giacobini et al. (2014) Djerbal et al. (2019)
Sema3F	Homeostatic synaptic plasticity Hippocampal synaptic transmission Npn-2: maintenance and function of corticostriatal circuitry	Wang et al. (2017) Sahay et al. (2005) Assous et al. (2019)
Sema3G	Maintenance of hippocampal synaptic connectivity and retention of fear memories	Tan et al. (2019)
Sema4C	Maintenance of hippocampal synaptic connectivity and retention of fear memories	Simonetti et al. (2019)
Sema5A	Control of maturation of adult-born hippocampal granule cells	Duan et al. (2014)
Sema7A	Inhibition of hippocampal neurogenesis Specific re-innervation of sweet taste receptor cells Control of GnRH release in the pituitary circulation	Jongbloets et al. (2017) Lee et al. (2017) Parkash et al. (2015)

*GnRH, gonadotropin releasing hormone; SVZ, subventricular zone.*

## Semaphorins in CNS Disease

Given the variety of functions that Semaphorins have during formation and maintenance of neuronal connections, it is not unexpected that they have been implicated in neurodevelopmental or psychiatric disorders characterized by dysfunctional neuronal networks. In the following sections we will highlight recent evidence of the involvement of Semaphorins in CNS disease.

### Schizophrenia

Schizophrenia is a psychiatric disease characterized by hallucinations, delusions, disorganized or catatonic behavior, and confused speech. Most patients experience cognitive symptoms, such as deficits in working memory, executive functioning and attention. A genetic component is recognized as a central factor in the development of the disease. A number of genes, as well as the possibility of complex gene-gene interactions, have been implicated (Insel, 2010). Abnormalities of brain development are increasingly recognized as culprits in the insurgence of schizophrenia. In post-mortem studies of patients with schizophrenia, several brain abnormalities are found, including alterations in cortical thickness, reduced hippocampal volume and hippocampal neurogenesis, neuronal misalignment in cortex and hippocampus, and decreased density of dendritic spines in the prefrontal cortex (Garey et al., 1998; Glantz and Lewis, 2000; Wong and Van Tol, 2003; van Swam et al., 2012; Weissleder et al., 2019). These observations suggest that schizophrenia may arise from defects in neuronal migration and synaptic connectivity (Conrad and Scheibel, 1987; Weinberger, 1987; Murray, 1994; Waddington et al., 1998), including excessive synaptic pruning, particularly during adolescence, when usually the first symptoms appear (Feinberg, 1982; Keshavan et al., 1994). Changes in the expression of Semaphorins or their downstream effectors were hypothesized to be involved in the pathogenesis of schizophrenia. Sema3A expression is highly increased in the cerebellum (namely in the Purkinje cell layer) and prefrontal cortex of schizophrenia patients compared to control subjects (Eastwood et al., 2003; Gilabert-Juan et al., 2015). Moreover, transcripts for the Sema3A receptor PlxnA1 are downregulated (Gilabert-Juan et al., 2015). However, it is not clear whether there is also increased expression of Sema3A in PNNs. CSPGs in PNNs are reported to be diminished in the brain of subjects with schizophrenia, including the prefrontal cortex (Pantazopoulos et al., 2010, 2015). An altered expression is also found for other Semaphorins and plexins in the prefrontal cortex of schizophrenic patients, with transcripts for PlxnB1 and Sema4D being upregulated, and transcripts for Sema3D downregulated (Gilabert-Juan et al., 2015). Two studies reported an alteration in Sema6C levels in schizophrenic prefrontal cortex, although with contrasting results (Arion et al., 2010; Gilabert-Juan et al., 2015). The expression of members of the CRMP family has also been found to be altered in animal models of schizophrenia as well as in the human brain (Quach et al., 2015).

Genome-wide association studies found a significant association between single nucleotide polymorphisms (SNPs) in PlxnA2 gene and schizophrenia in patients with European, European-American, or Latin-American descent (Mah et al., 2006). Follow-up studies expanded these findings to a Japanese population (Takeshita et al., 2008). However, other studies found no significant association between SNPs in PlxnA2 and schizophrenia in Japanese or Chinese populations (Fujii et al., 2007; Budel et al., 2008). This suggests that, in different populations, PlxnA2 may confer varying genetic risk to schizophrenia. SNPs in PlxnA2 may induce changes in PlxnA2 conformations, which in turn may influence its cellular localization, function, or affinity for its ligands [Sema3B, Sema5A, Sema6A (Renaud et al., 2008; Sabag et al., 2014; Zhao et al., 2018)]. Interestingly, mice deficient for PlxnA2 show defective hippocampal neurogenesis and impairments in sociability, associative learning and sensorimotor gating, which are traits commonly observed in schizophrenia patients (Zhao et al., 2018).

It is not clear whether changes in the expression levels of Semaphorins or molecules of their signaling pathway play a causal role in schizophrenia onset/progression or are simply a consequence of the disease. However, a link between PlxnA2 mutation and schizophrenia has been found in both humans and mouse models, pointing to an involvement of PlxnA2 signaling in the etiology of schizophrenia. We can speculate that impaired PlxnA2 signaling may be responsible for circuits defects that are typically found in the schizophrenic brain. However, further studies in which the expression of PlxnA2 or its ligands is modulated spatio-temporally, for instance by employing inducible conditional knock-out mice, may help elucidate this issue.

### Anxiety and Depression

Anxiety and depression are among the major causes of disability worldwide. Defective hippocampal neurogenesis is suggested to facilitate the development of anxiety and depression (Jacobs et al., 2000), and recent data indicate that the effect of antidepressants depends on their ability to induce hippocampal neurogenesis (Kong et al., 2009; Mateus-Pinheiro et al., 2013). As seen in the previous section, PlxnA2 deficiency in mice leads to altered hippocampal neurogenesis, as well as schizophrenia-like traits (Zhao et al., 2018). When testing the hypothesis that PlxnA2 might be associated with other psychiatric conditions, Wray et al. (2007) found evidence of an association between a SNP in plxnA2 gene and anxiety, as well as depression, neuroticism, and psychological distress, particularly in individuals who were comorbid for anxiety. This suggests that variants of PlxnA2 may play a causal role in anxiety disorders.

An additional indication that semaphorin signaling may be implicated in the development of anxiety comes from the study by Matsuda et al. (2016) on mice deficient for Sema3F. These mice show anxiety-related behaviors in novel environments, as demonstrated by increased latency to enter the light chamber in the light/dark transition test, reduced time spent in the center area in the open field, and decreased locomotor activity in the elevated plus maze, when compared to wild-type mice. In addition, in the social interaction test, which has also been used to assess anxiety (File and Seth, 2003), Sema3F knock-out mice show reduced duration of active social contact with a stranger mouse compared with controls (Matsuda et al., 2016).

Alcohol dependence and depression are frequently comorbid, although causal links between the two disorders are unknown (Grant and Harford, 1995). Interestingly, genome-wide association studies identified a risk variant in the sema3A gene in African American participants. No association was detected in this gene in European American participants, indicating a population-specific genetic risk (Zhou et al., 2017). Sema3A risk locus was not identified in genome-wide association analysis of either disease separately, maybe due to the small sample size used. The specific contribution of Sema3A mutations to the possible causes of alcohol dependence and depression remains to be elucidated.

Overall, only a few studies have implicated semaphorin signaling in psychiatric disorders such as anxiety and depression. Although a role of PlxnA2 has been demonstrated in anxiety, from association results it is not possible to determine whether the putative functional role of PlxnA2 takes place during development or in the adult brain (possibly acting on mechanisms regulating neurogenesis). Further research on mutant mice may help clarify the involvement of Semaphorins/plexins in psychiatric diseases.

Depression is often associated with structural abnormalities within specific neuronal networks (Ressler and Mayberg, 2007; Price and Drevets, 2010), raising the possibility that changes in information processing, rather than a deficiency in monoaminergic neuromodulators, are a key component of this condition. Indeed, despite fast drug-induced elevations of monoamine levels, symptom improvement requires weeks of antidepressant treatment. Recent evidence suggests that recovery from depression is based on structural and functional changes in critical neuronal networks (Castrén and Hen, 2013). The antidepressant fluoxetine has been shown to restore juvenile-like plasticity in the adult brain (Maya-Vetencourt et al., 2008; Karpova et al., 2011). Therefore, antidepressants may promote reorganization of neuronal networks (Lesnikova et al., 2021), which, guided by activity, would allow them to better adapt to environmental conditions. In this framework, Sema3A or other plasticity inhibitors of the Semaphorin family may be interesting targets to treat depression.

### Epilepsy

Epilepsy is a chronic neurological disease characterized by spontaneous recurrent seizures. Seizures are due to synchronous firing of neurons in the CNS, which results from an imbalance between GABAergic and glutamatergic neurotransmission. Seizures can be focal, affecting only a discrete part of the brain, or generalized, encompassing larger brain regions in both hemispheres. Mesial temporal lobe epilepsy (TLE), one of the most common forms of epilepsy, is characterized by deficits in memory, language, attention, and executive functions. The main causes of epilepsy are genetic variants in neural genes, brain insults, infections, and developmental malformations. Inherited forms of epilepsy account for 20% of all epilepsies (Shin and McNamara, 1994). Nonetheless, following brain injury, the genetic background of an individual is likely to affect the probability of insurgence of epilepsy. A genetic predisposition for injury-induced epilepsy is evident in mice. While the FVB/NJ mouse strain develops permanent epilepsy following neuronal injury induced by kainic acid, the C57Bl/6J mouse strain does not (Copping et al., 2019).

A sequela of molecular and cellular alterations (e.g., changes in ion channel activity, post-translational changes to neurotransmitter receptors, induction of immediate early genes) underlie epileptogenesis. These alterations are followed by chronic anatomical changes, including mossy fiber sprouting, network reorganization, and gliosis in the hippocampus (Rakhade and Jensen, 2009). Because Semaphorins are involved in many steps of neuronal networks development, they have been studied as potential candidates in the etiology of epilepsy. Sema3F was identified as a gene that is downregulated in hippocampal pyramidal cells of FVB/NJ mice (which are epileptogenic sensitive) but not in C57Bl/6J mice (which are epileptogenic resistant) following kainic acid induction (Yang et al., 2005). The expression of other members of the Semaphorin family (e.g., Sema3A, Sema4C) remains unchanged, demonstrating that kainic acid does not generally effect the expression of Semaphorins in the brain (Yang et al., 2005). In addition, Sema3F knockout mice are more prone to seizures than wild-type animals, even in the absence of neuronal injury (Sahay et al., 2005). Anatomic and electrophysiologic studies have demonstrated the presence of anomalous recurrent excitatory synapses among dentate granule cells as well as CA1 pyramidal cells in models of TLE (Perez, 1996; Wuarin and Dudek, 1996; Esclapez et al., 1999). Because secretion of Sema3F by CA1 pyramidal cells may constrain axonal sprouting, reduced levels of Sema3F after induced status epilepticus may favor axonal remodeling and synapse reorganization, which are likely to provoke seizures. Similarly, the expression of Sema3F protein in the dentate gyrus is decreased in a lithium-pilocarpine-induced status epilepticus mouse model, in parallel to mossy fiber sprouting occurring in that region, suggesting that reduced expression of Sema3F may facilitate anomalous growth of mossy fibers (Cai et al., 2016). Experiments in rats confirmed a reduction in the expression of Sema3F mRNA in the CA1 and CA3 regions of the hippocampus following kainic acid injection (Barnes et al., 2003).

The aberrant development of GABAergic circuitry is a possible risk factor in epilepsy. Interestingly, a knockout of the Sema3F gene specifically in interneurons results in a reduced number of interneurons, decreased interneuron neurite outgrowth, and increased excitability, which are accompanied by spontaneous seizures. Elevated levels of antigens of oxidative stress, inflammation, and microglia activation are also found in Sema3F knock-out mice, suggesting that Sema3F signaling in the immune system may affect the developing brain (Li et al., 2019). Notably, decreased numbers of interneurons have been found in the temporal lobe of patients with epilepsy and animal models of epilepsy (Spreafico et al., 2000; André et al., 2001; Sundstrom et al., 2001). In accordance with the abovementioned evidence, mutant mice for Npn-2 (which is a Sema3F receptor) show hippocampal wiring defects and develop seizures (Giger et al., 2000), and a deficit in Npn-2 during development induces a reduction in dendritic length and complexity and spine numbers on CA1 pyramidal neurons, as well as decreased survival of many types of interneuron, which result in spontaneous recurrent seizure activity after chemical challenge (Gant et al., 2009).

Although dysregulation of Semaphorins in epileptic humans has not yet been reported, the mRNA encoding Sema3F is a target of the fragile X mental retardation protein (FMRP) and is decreased in polysomes from fragile X syndrome patients’ cells, suggesting that Sema3F is downregulated in these patients (Darnell et al., 2001). The knockout mouse of Fmr1, the gene that encodes for FMRP, demonstrates defects in the mossy fiber infrapyramidal tract that are similar to those observed in the Sema3F, Npn-2, and PlxnA3 knockout animals, suggesting that regulation of Sema3F by FMRP may be important for the correct wiring of the hippocampus (Ivanco and Greenough, 2002). Interestingly, 10–20% of individuals with the fragile X syndrome also develop epilepsy (Berry-Kravis, 2002), raising the possibility that dysregulation of Sema3F predisposes humans to epilepsy as well.

Changes in expression levels of other Semaphorins have been also documented in animal models of epilepsy (Barnes et al., 2003; Holtmaat et al., 2003). Neurons of layer II of the adult entorhinal cortex (stellate cells) express Sema3A mRNA (Giger et al., 1998). Stellate cells project to the molecular layer of the dentate gyrus, where they may secrete Sema3A. After induction of status epilepticus, a downregulation of Sema3A mRNA in the entorhinal cortex has been observed concomitantly with an upregulation of mRNA for the growth-associated protein GAP-43 in granule cells. At later time points, mossy fibers vigorously sprout into the dentate gyrus molecular layer (Gorter et al., 2001, 2002). These results suggest that a reduction of Sema3A protein in the molecular layer would allow the growth of mossy fibers during epilepsy.

Sema3A is abundantly expressed in PNNs. Interestingly, PNNs are decreased in the hippocampus of animal models of epilepsy, possibly due to altered expression of PNN degrading enzymes (Mcrae et al., 2012; Rankin-Gee et al., 2015). As a consequence, Sema3A protein may be displaced from the synapses and this may favor aberrant neurite outgrowth and synapse formation on PNN-bearing neurons.

Recent evidence shows that the expression of CRMP-1 and -2 is strongly decreased in the temporal cortex of TLE patients (Czech et al., 2004; Luo et al., 2012). In a rat pilocarpine-induced epilepsy model, which is characterized by mossy fiber sprouting and spontaneous seizure generation (Shibley and Smith, 2002), CRMP-1 labeling in CA1 and CA3 pyramidal cells and adjacent neocortex is decreased (Luo et al., 2012). The reduction of CRMP proteins may contribute to the formation of recurrent excitatory networks in TLE.

Overall, studies in knock-out mice suggest that Sema3F signaling may be implicated in the etiology of epilepsy. Sema3F downregulation may induce changes in neuronal wiring, resulting in seizures. However, it is still unclear if changes in expression of other Semaphorins, such as Sema3A, may play a causative role in the onset of the disease or occurs as a consequence of the disease. Nonetheless, downregulation of Semaphorin signaling induced by seizures may cause changes in neuronal circuitry, which may result in further seizures and, thus, exacerbation of disease symptoms.

### Autism

Autism spectrum disorder (ASD) is a neurodevelopmental syndrome characterized by repetitive behaviors and deficits in social skills and language. Although the etiology of ASD is yet unclear, in the majority of the cases (50–90%) it is thought to be genetic. A unifying model proposes that ASD is the consequence of aberrant developmental wiring of brain regions that are involved in higher-order functions (Kelleher and Bear, 2008). Indeed, a growing number of ASD-associated genes encode synaptic proteins (Peça and Feng, 2012; Voineagu and Eapen, 2013). Interestingly, several Semaphorins have been linked to ASD. Weiss et al. (2008) identified microdeletions and microduplications of chromosome 16p11.2 that carry substantial susceptibility to ASD, accounting for approximately 1% of cases. One of the genes from the affected region encodes for TAOK2 (thousand-and-one-amino acid 2 kinase), a member of the MAP kinase family that interacts with Npn-1. Sema3A induces TAOK2 phosphorylation, thereby activating it. TAOK2, in turn, modulates the Sema3A–Npn-1 pathway that controls basal dendrite arborization of cortical pyramidal neurons (Fenstermaker et al., 2004). It has been shown that TAOK2 downregulation impairs the formation of basal dendrites, whereas TAOK2 overexpression restores deficits in basal dendrite formation induced by inactivation or knock-out of Npn-1 (de Anda et al., 2012). This suggests that loss-of-function of TAOK2 may affect Sema3A-mediated regulation of dendritic formation, leading to abnormal development of the cortical network.

Semaphorins dysfunction has also been linked to Rett syndrome (Degano et al., 2009), an autism spectrum disorder that results from mutations in the transcriptional regulator methyl-CpG binding protein 2 (MECP2; Ip et al., 2018). Mouse models lacking MeCP2 or expressing a mutant form of MeCP2 share many features of the human disorder (Chen et al., 2001; Shahbazian et al., 2002; Pelka et al., 2006). By employing these mice, Degano et al. (2009) observed severe defects in axonal guidance in the developing olfactory system, and altered expression levels of components of the Sema3F–Npn-2–PlxnA3 and Sema3A–Npn-1–PlxnA4 pathways, suggesting that MeCP2 controls the expression of Sema3A and Sema3F and their receptors during development. Indeed, olfactory axons from Mecp2 mutant mice display reduced repulsion when co-cultured with mutant olfactory bulb explants, but not with wild-type olfactory bulbs (Degano et al., 2009). Thus, time and space-dependent transcriptional dysregulation of Semaphorins and/or their receptors could account for defects in the development of neural connectivity caused by Mecp2 mutation.

Finally, both Sema3F and Npn-2 null mice recapitulate some aspects of autistic behaviors (Shiflett et al., 2015; Matsuda et al., 2016). In addition, mice in which Npn-2 is selectively knocked-out in interneurons show neuropathological traits similar to those found in ASD brains (Gant et al., 2009). Similarly, interneuron-specific knockout mice of Sema3F display reductions in sociability and increased repetitive behaviors (Li et al., 2019).

In a genome wide association study, Sema5A has been identified as ASD susceptibility gene (Weiss et al., 2008). Moreover, Sema5A expression is found to be reduced in Brodmann area 19 as well as in lymphocytes of autistic subjects (Melin et al., 2006; Weiss et al., 2008). Sema5A^–/–^ mutants exhibit several alterations, from deficits in sociability, to exuberant excitatory synapses and increased excitatory synaptic transmission in dentate granule cells (Duan et al., 2014). Additional studies are needed to determine the cellular basis of the behavioral deficits observed in Sema5A^–/–^ mice. Studies utilizing Sema5A conditional mutants that lack Sema5A in specific neural cell-types or specific brain structures may help identify where Sema5A function is required for proper neural circuit development. Additional evidence that Sema5A dysfunction could lead to ASD comes from the study by Mosca-Boidron et al. (2016), which reports a *de novo* translocation in the Sema5A gene, associated with a partial deletion, in a patient with ASD.

### Multiple Sclerosis

Multiple sclerosis (MS) is a CNS disease characterized by multifocal inflammation and immune-mediated damage to myelin sheaths, which disrupts axonal signal conduction. As demyelinated axons are prone to injury and degeneration, various degrees of axonal damage and neurodegeneration occur in MS patients, which contribute to MS progression and permanent disability involving motor and cognitive functions (Compston and Coles, 2008). OPCs are the main source of remyelinating cells. Interestingly, in active demyelinating lesions in human MS brains, numerous glial cells, including OPCs, express transcripts for Sema3A and Sema3F as well as Npn, which are normally not detected in the intact white matter (Williams et al., 2007). Moreover, an increased number of PlxnA1 expressing oligodendrocytes is found in the white matter of MS patients (Binamé et al., 2019). Because Semaphorins are known inhibitors of axon regeneration (Mecollari et al., 2014), in MS lesions they may hinder the attempts of lesioned, but also intact neurons to reorganize their connections in response to injury. Expression levels of Sema3A and 3F are also increased in neurons projecting to the lesion site (Williams et al., 2007), suggesting that Semaphorin upregulation in neurons may be a consequence of axon demyelination or a response to axonal insult/degeneration. On the other hand, since Sema3A and Sema3F are expressed in OPCs during white matter development, where they act as OPC chemorepulsive or chemoattractant cues, respectively (Sugimoto et al., 2001; Spassky et al., 2002; Tsai and Miller, 2002), they may influence OPC recruitment toward demyelinated lesions. Sema3F expressing cells are particularly abundant in MS lesions characterized by strong inflammation, suggesting that inflammatory lesions are associated with higher levels of OPC attractive cues to promote OPC migration and, as a consequence, myelin repair. On the contrary, Sema3A is more abundant in less inflammatory lesions, where myelination is lower, suggestive of a chemorepulsive role of Sema3A on OPC migration (Williams et al., 2007). Indeed, lentiviral-mediated overexpression of Sema3F in demyelinated areas induces an increase in the number of OPCs as well as remyelination. Overexpression of Sema3A results instead in decreased OPC recruitment, while an opposite effect is found by Sema3A loss-of-function experiments (Piaton et al., 2011). When Sema3A is administered to an oligodendroglial precursor cell line or cultured NSCs, cell migration and expression of myelin basic protein are strongly reduced, reinforcing the notion that Sema3A inhibits both OPC differentiation and oligodendrocyte migration (Binamé et al., 2019). Moreover, following infusion of Sema3A into demyelinated rat cerebellar peduncle, OPC differentiation and remyelination are strongly inhibited although the number of OPCs in demyelinated lesions is not altered (Syed et al., 2011). Interestingly, injection of a peptide inhibiting Sema3A signaling (by antagonizing Npn-1-PlxnA1 dimerization) is able to induce myelin recovery and rescue motor deficits in mouse models of MS (Binamé et al., 2019). The combination of anti-inflammatory drugs (which are current treatments for MS) with therapies aiming at inhibiting Sema3A signaling in order to repair myelin and enhancing neuronal plasticity would be an interesting strategy toward a regenerative treatment for MS.

Other Semaphorins have been recently implicated in MS. Sema7A has been detected in neurons close to MS lesions as well as in reactive astrocytes and oligodendrocytes in the damaged white matter in mice and human tissue (Costa et al., 2015; Gutiérrez-Franco et al., 2016), suggesting it may have an inhibitory role in compensatory axonal remodeling in lesioned areas. Moreover, Sema7A is upregulated in mice in inflammatory cells infiltrating the CNS and in blood immune cells during the inflammatory phase of experimental autoimmune encephalomyelitis (EAE, a widely accepted model of MS), suggestive of a role in the immune response (Gutiérrez-Franco et al., 2016). Indeed, microglia and macrophages express Sema7A receptors (Costa et al., 2015) and Sema7A induces a strong activation of monocytes and macrophages as well as production of pro-inflammatory cytokines (Holmes et al., 2002). Moreover, in Sema7A knock-out mice with EAE, the disease is milder than in wild-type mice, suggesting that Sema7A is involved in peripheral immunity and inflammation during MS (Gutiérrez-Franco et al., 2016, 2017).

Furthermore, Sema4D participates in several processes which are compromised during MS, including migration and differentiation of OPCs, immune cell regulation and blood brain barrier integrity (Giraudon et al., 2004, 2005; Suzuki et al., 2008; Yamaguchi et al., 2012). Inhibiting Sema4D activity by anti-Sema4D antibodies that block the interaction of Sema4D with its receptors results in improvement of clinical scores in EAE-rodents and in enhanced myelin integrity. *In vitro* experiments show that administering Sema4D to OPCs decreases their differentiation, and this effect is reversed by anti-Sema4D antibodies (Smith et al., 2015). At present a clinical trial is ongoing to test the potential of the antibodies-based inhibition of Sema4D as a novel therapeutic strategy for MS, and the phase I has been successfully completed (LaGanke et al., 2017).

### Amyotrophic Lateral Sclerosis

Amyotrophic lateral sclerosis (ALS) is a fatal neurodegenerative disease. It is characterized by gradual degeneration of motoneurons in the brain and spinal cord, leading to progressive paralysis of skeletal muscles and death within 5 years of diagnosis due to respiratory failure. The vast majority of ALS cases (90%) are considered sporadic, with various genetic or environmental factors influencing the disease. Fast-fatigable motoneurons are the most vulnerable in ALS. The mechanisms leading to motoneuron death are not yet completely elucidated. However, the observation that the first pathophysiological changes observed in patients occur at the NMJ has given rise to the theory that ALS is a distal axonopathy, caused by alterations in skeletal muscles, muscle satellite cells or terminal Schwann cells, before motoneuron degeneration occurs (Moloney et al., 2014). Interestingly, aberrant expression or function of axon guidance cues, including Semaphorins and ephrins, have been recently proposed to be linked to the pathogenic mechanism of ALS. Sema3A is upregulated in terminal Schwann cells at the NMJ in type IIb muscle fibers (which are innervated by fast-fatigable motoneurons) in pre-symptomatic ALS mice, suggesting that it may induce the retraction of these terminals from the neuromuscular synapse (de Winter et al., 2006). Moreover, increased Sema3A expression has been found in cortical motoneurons of ALS patients, where it may cause axonal degeneration or prevent regeneration of motor axons (Korner et al., 2016). Notably, interfering with Sema3A-Npn-1 binding in adult motoneurons leads to improved motor functions and survival of ALS mice (Moloney et al., 2014; Venkova et al., 2014). In contrast, in ALS mice with chronically diminished Sema3A signaling, the decline in motor functions is not improved (Moloney et al., 2017). However, in the latter case, processes compensating the chronic defect in Sema3A signaling may mask the role of Sema3A in ALS. In presymptomatic ALS mice, CRMP4a is upregulated in a subset of lumbar motor neurons, and overexpression of CRMP4a leads to degeneration of 30% of spinal motoneurons (Duplan et al., 2010). In human patients, a missense mutation within the CRMP4a gene has been found in a French population, and overexpression of CRMP4a protein bearing such mutation in motoneurons *in vitro* accelerates cell death through a detrimental effect on axonal growth (Blasco et al., 2013). CRMP4 may mediate Sema3A effects on ALS pathology, as Sema3A may signal through CRMP4 (Niisato et al., 2012).

Furthermore, Birger et al. (2018) demonstrated the ability of Sema3A to reduce cell survival of human cortical neurons and, in contrast, to stimulate neuronal survival of human spinal motoneurons. These observations are consistent with the upregulation of Sema3A in the cortex of ALS patients (Korner et al., 2016), suggesting that this protein may be a contributing factor in the loss of neurons in the cortex of ALS patients.

Interestingly, astrocytes are involved in ALS progression in mice (Yamanaka et al., 2008), and patient-derived astrocytes are toxic toward wild-type motoneurons *in vitro* (Haidet-Phillips et al., 2011; Meyer et al., 2014). Astrocytes regulate many neuronal functions including axon maintenance, and part of this communication is regulated through secreted extracellular vesicles (EVs; Frühbeis et al., 2013). Specifically, EV miRNA cargo can modulate neuronal and astrocytic function in health and disease (Chaudhuri et al., 2018). Varcianna et al. (2019) showed that induced astrocytes derived from human fibroblasts secrete miRNAs regulating transcripts for proteins involved in axonal growth and maintenance. Notably, EVs isolated from the conditioned medium of induced astrocytes derived from fibroblasts of ALS patients are sufficient to cause motoneuron death even in presence of trophic factors, demonstrating that EVs carry toxic factors. Moreover, conditioned medium from ALS astrocytes causes axonal suffering before motor neuron cell body loss. In conditioned medium derived from ALS astrocytes, there is a significant downregulation of a miRNA (miR-494-3p) involved in the regulation of several genes, including the inhibition of Sema3A expression. Treatment of cultured mouse motoneurons with conditioned medium from ALS astrocytes supplemented with miR-494-3p reduces Sema3A levels, rescues neurite length and motor neuron survival (Varcianna et al., 2019).

Additionally, Sema3A may be involved in the myogenic program necessary for muscle regeneration after muscle injury. Namely, Sema3A, which is expressed by satellite cells after muscle injury or denervation (Do et al., 2011), may be beneficial for skeletal muscle regeneration by delaying neuronal sprouting and re-attachment of nerve terminals until damaged muscle fibers have been repaired. Thus, it is possible that, upon ALS-related denervation, satellite cells begin to produce Sema3A. The myogenic pathway is indeed active in presymptomatic ALS mice, but the function of satellite cells becomes impaired as ALS progresses (Pradat et al., 2011; Manzano et al., 2013) and levels of myogenic proteins decrease (Manzano et al., 2011). If the myogenic process is unable to maintain muscle regeneration, muscle fibers are not restored and Sema3A may keep on delaying the re-attachment of terminals, contributing to the progression of muscle weakening and paralysis.

Based on this bulk of evidence, we can conclude that hyperactive Sema3A signaling may be the leading cause of axonal degeneration and motoneuron death in ALS, making Sema3A a potential therapeutic target in this disease.

### Alzheimer’s Disease

Alzheimer disease (AD) is the most common age-related dementia, characterized by progressive degeneration of neurons in the neocortex and hippocampus (Braak and Braak, 1991; Price et al., 1991).

Alzheimer disease is divided into familial AD and sporadic AD (Dorszewska et al., 2016). The vast majority of AD is sporadic, and is caused by a combination of genetic and environmental risk factors. Only 5% of the AD cases are familial, which might be caused by autosomal mutations in β−amyloid precursor protein, presenilin 1 and/or presenilin 2 (Waring and Rosenberg, 2008). AD is characterized by extraneuronal deposition of amyloid β (Aβ) protein in the form of plaques and intraneuronal aggregation of microtubule-associated protein tau in the form of filaments. Braak et al. (1994) proposed that abnormal tau phosphorylation is a crucial step leading to the formation of tau filaments, and that, unlike Aβ accumulation, the spread of tau filaments is associated with the clinical progression of AD. Interestingly, immunohistochemical studies performed by Good et al. (2004) in adult human brains reveal that Sema3A shows a punctate pattern on the membrane of neurons (although it is not clear if it is associated with perineuronal nets), whereas in AD patients individual neurons display either punctate surface labeling staining or granular intracellular labeling. Interestingly, at the onset of neurofibrillary tangle formation the majority of CA1 neurons labeled for Sema3A are devoid of neurofibrillary tangles, suggesting that Sema3A accumulation may precede tau phosphorylation during the development of AD. Later on during the disease process, accumulation of Sema3A is found to colocalize with phosphorylated tau and microtubule associated protein 1B (MAP1B) in many neurons. Neurons responding to Sema3A may activate kinases that promote phosphorylation of tau. Although a number of kinases have been shown to phosphorylate tau *in vitro*, the key players *in vivo* are GSK-3b and Cdk5 (Lew et al., 1994; Lovestone et al., 1994; Wagner et al., 1996; Patrick et al., 1999; Lau et al., 2002), and these kinases have been suggested to mediate the functions of Sema3A (Eickholt et al., 2002). Sustained activation of Cdk5 and GSK-3, or inhibition of phosphatase activities (Bennecib et al., 2000; Planel et al., 2001), may result in pathogenic hyperphosphorylation of tau and MAP1B during AD. Altogether, degeneration of neurons in the CA1 during the early stages of AD may be caused by aberrant Sema3A signaling following intracellular accumulation of Sema3A, which may contribute to the acceleration of tau phosphorylation, leading to neurofibrillary tangle formation (Good et al., 2004). In this respect, phosphorylated CRMP2 protein has been observed in neurofibrillary tangles in the brain of AD patients, suggesting that phosphorylation of CRMP, activated by Sema3A, may be relevant for the pathological aggregations of microtubule-associated proteins (Uchida et al., 2005).

In addition, since Sema3A can directly induce neurodegeneration and apoptosis of neural progenitor cells (Bagnard et al., 2001), sensory neurons (Gagliardini and Fankhauser, 1999), cerebellar granule cells and sympathetic neurons *in vitro* (Shirvan et al., 1999, 2000), it may play a direct role in neurodegeneration in AD.

From a genetic perspective, two SNPs in Sema3A gene, which lead to an amino acid substitution, were deposited in the genome browser ‘‘Ensemble’’^1^, but they were not detected in an Italian population of AD patients, suggesting that Sema3A does not act as risk factor toward the development of AD (Villa et al., 2010). On the contrary, a significant association between AD risk and SNPs in PlxnA4 has been recently identified, and higher levels of PlxnA4 isoforms in cortical brain tissue were observed in late stage AD cases compared to controls, which were significantly correlated with the clinical dementia rating score, plaque density, and Braak stage (Jun et al., 2014). In addition, by using RNA sequence data from AD patients and building a molecular network using modules of coexpressed genes, PlxnB1 gene has been found to be strongly correlated with β-amyloid burden as well as cognitive decline in older individuals, and with extracellular β-amyloid levels in astrocyte cultures (Mostafavi et al., 2018).

Based on the data showing a correlation between Semaphorins/Plxns and AD pathology, it would be interesting to investigate whether targeted overexpression of Semaphorins, such as Sema3A, or Plxns, such as PlxnA4 or PlxnB1, in the hippocampus or cortex of mice is sufficient to induce an AD-like phenotype.

In order to overcome the progressive loss of functional connections due to neurodegeneration in AD, new neuronal connections may help bypass non-functional neurons, leading to functional improvements. In order to increase axonal plasticity to compensate for neuronal loss in AD, interfering with Sema3A in PNNs may be an interesting path to explore. In this context, enzymatic digestion of PNN-CSPGs in the perirhinal cortex of AD mice with neurodegenerative tauopathy results in restoration of normal synaptic transmission and memory improvement (Yang et al., 2015).

## Discussion

Semaphorins regulate several processes during nervous system development, from cell proliferation, differentiation and migration to neuritogenesis and synapse formation. In recent years it has become increasing clear that Semaphorins are also pivotal molecules in the control of structure and function of neural circuits throughout life. However, it is not known whether they employ similar molecular mechanisms throughout different stages of development and in the adulthood. It is possible that specific Semaphorin downstream signaling pathways are employed for the execution of specific functions, depending on neuronal cell type, neuronal compartment (growth cone, synapse, dendrites, etc.), and age.

Interestingly, Semaphorin expression is altered in several disorders characterized by neuronal circuits alterations (Table 2). However, in many instances it is difficult to discriminate between a causal role of Semaphorins in the disease and a change in expression occurring during the disease process. In the latter case, however, Semaphorins may amplify the severity of the disease. Furthermore, it is complicated to distinguish between developmental and adult effects of Semaphorin alterations on a disease. Employing mutant mice in which Semaphorin expression is spatio-temporally regulated may help elucidate those issues.

**TABLE 2:** Overview of the involvement of Semaphorins or their receptors in neuropsychiatric/neurological diseases.

Disease	Human mutation	Expression changes in human brain	Mutant mice that develop the disease or modulation of gene expression in mice
Schizophrenia	PlexinA2	↑ Sema3A ↑ Sema4D ↑ PlxnB1 ↓ PlxnA1 ↓ Sema3D	PlxnA2 -/- mice
Anxiety	PlexinA2	–	Sema3F -/- mice
Depression (comorbidity with alcohol dependence)	Sema3A	–	–
Epilepsy	–	↓ CRMP-1 ↓ CRMP-2	Sema3F -/- mice Npn-2 -/- mice
Autism	Sema5A	↓ Sema5A	Sema5A -/- mice Sema3F -/- mice Npn-2 -/- mice
Multiple sclerosis	–	↑ Sema3A ↑ Sema3F ↑ Sema4D ↑ Sema7A ↑ Npn ↑ PlexinA1	Milder symptoms/increased remyelination: - In Npn-1 -/- mice; - In Sema7A -/- mice; - After inhibition of Sema3A signaling; - After overexpression of Sema3F; - After infusion of anti-Sema4D antibodies More severe symptoms/decreased remyelination: - After overexpression of Sema3A
ALS	–	↑ Sema3A	–
Alzheimer’s disease	–	↑ PlexinA4 ↑ PlexinB1	–

Overall, Semaphorins and their associated receptors and signaling proteins may represent valuable biomarkers for monitoring disease progression as well as promising therapeutic targets for treating debilitating brain diseases.

## Author Contributions

All authors contributed to the article and approved the submitted version.

## Conflict of Interest

The authors declare that the research was conducted in the absence of any commercial or financial relationships that could be construed as a potential conflict of interest.

## Footnotes

1 http://ensembl.org/Homo_sapiens
